# High-quality Japanese flounder genome aids in identifying stress-related genes using gene coexpression network

**DOI:** 10.1038/s41597-022-01821-5

**Published:** 2022-11-16

**Authors:** Xi-wen Xu, Weiwei Zheng, Yingming Yang, Jilun Hou, Songlin Chen

**Affiliations:** 1 Laboratory for Marine Fisheries Science and Food Production Processes, Qingdao National Laboratory for Marine Science and Technology, Yellow Sea Fisheries Research Institute, Chinese Academy of Fishery Sciences, Nanjing Road 106, Qingdao, 266071 China; 2 Key Lab of Sustainable Development of Marine Fisheries, Ministry of Agriculture and Rural Affairs, Wenhai Road 1, Qingdao, 266071 China; 3 College of Fisheries and Life Science, Shanghai Ocean University, Shanghai, China; 4 Beidaihe Central Experiment Station, Chinese Academy of Fishery Sciences, Qinhuangdao, Hebei 066100 China

**Keywords:** Transcriptomics, Gene expression profiling

## Abstract

The Japanese flounder is one of the most economically important marine flatfish. However, due to the increased frequency of extreme weather events and high-density industrial farming, an increasing number of environmental stresses have become severe threats to the healthy development of the Japanese flounder culture industry. Herein, we produced a high-quality chromosome-scale Japanese flounder genome using PacBio Circular Consensus Sequencing technologies. The assembled Japanese flounder genome spanned 588.22 Mb with a contig N50 size of 24.35 Mb. In total, 105.89 Mb of repetitive sequences and 22,565 protein-coding genes were identified by genome annotation. In addition, 67 candidate genes responding to distinct stresses were identified by gene coexpression network analysis based on 16 published stress-related RNA-seq datasets encompassing 198 samples. A high-quality chromosome-scale Japanese flounder genome and candidate stress-related gene set will not only serve as key resources for genomics studies and further research on the underlying stress responsive molecular mechanisms in Japanese flounder but will also advance the progress of genetic improvement and comprehensive stress-resistant molecular breeding of Japanese flounder.

## Abstract (summary)

**Table:** 

Measurement(s)	genome assembly
Technology Type(s)	PacBio RS II

## Background & Summary

Japanese flounder (*Paralichthys olivaceus*, FishBase ID: 1351), naturally distributed in the Western Pacific: Korean Peninsula, Kuril Islands, Japan, Sakhalin to the South China Sea, is now an economically important cosmopolitan marine-culture flatfish species due to its delicious taste and high nutritional value^1^. In recent years, however, Japanese flounder have suffered from an increasing number of environmental stresses, such as the increased temperature of global water^2^, a wide range of environmental pollutants derived from agricultural and industrial wastes^3–5^, and the outbreak of fish diseases caused by high-density industrial farming^6^, which have severely threatened the healthy development of the Japanese flounder industry and led to enormous economic losses. Hence, acquiring a high-quality chromosome-scale assembled genome of Japanese flounder and obtaining abundant gene resources associated with stress resistance are necessary, which will aid in research on the molecular mechanism of stress resistance in Japanese flounder and provide theoretical support for the subsequent genetic improvement of Japanese flounder germplasm.

To the best of our knowledge, a high-quality chromosome-scale assembled genome is the basis of genetic resource mining and is critical for efficient molecular breeding. To date, two genome assemblies of Japanese flounder including one from our own group have been published in the National Center for Biotechnology Information (NCBI)^7,8^. However, because of the restriction of sequencing techniques and assembly algorithms, the genome assemblies remain fragmented. Therefore, it is urgent to obtain an improved high-quality Japanese flounder genome. The long-read and high-accuracy PacBio single molecule sequencing technique (SMRT) and the emergence of corresponding advanced assembly methods make it possible.

RNA-sequencing (RNA-seq) technology has now become an effective tool for obtaining large-scale genetic information and functional genes, which has promoted the exploration of molecular mechanisms of resistance. Recently, many RNA-seq studies of Japanese flounder have made significant progress in detecting stress responsive genes and molecular mechanisms under various environmental stresses, such as chemical stress^3,5,9,10^, heat stress^11,12^, and pathogen stress^6,13–18^. Although many key pathways and genes have been identified during environmental stresses through conventional transcriptome profiling, little attention has been given to connectivity analysis between genes by complex gene coexpression networks (GCNs). In this study, we reanalyzed multiple RNA-seq datasets from Japanese flounder using GCN analysis, which can explore the system-level functionality of the transcriptome^19^. GCN analysis can reveal the interrelation between gene modules and traits and identify key modules and the most central and vital hub genes, which provides an efficacious method to explore the in-depth mechanism of complex traits^20^. GCN analyses have been used extensively to identify key gene modules and genes in various teleosts, such as *Scophthalmus maximus*^21,22^ and *Nibea albiflora*^23^.

In the present study, we assembled a high-quality chromosome-scale Japanese flounder genome, of which the contig N50 size reached 24.35 Mb, using PacBio High fidelity (HiFi) reads. Based on this high-quality genome assembly, we further improved the annotation of the repetitive sequences and protein-coding genes in Japanese flounder. Moreover, we reanalyzed all published stress-related RNA-seq datasets from Japanese flounder from the NCBI Sequence Read Archive (SRA) database using GCN analysis, and identified multiple gene modules and candidate genes that respond to various environmental stresses in Japanese flounder. In summary, our study produced abundant and valuable genetic resources at the genome and gene levels, which can be used for further research on the functional genome and stress response mechanisms of Japanese flounder and can also provide theoretical support for the development of molecular breeding technology for resistant Japanese flounder varieties.

## Methods

### Japanese flounder samples and genome sequencing

Genomic DNA was isolated from fresh muscle samples of a female Japanese flounder. The degree of DNA degradation and RNA protein contamination were analyzed using 1% agarose gel electrophoresis, and the concentration was accurately quantified using Qubit.

Then, qualified genomic DNA was sheared into fragments, and the size-selection of fragments was performed using BluePippin. After the two ends of size-selected fragments were repaired, a poly-A tail and ligated adaptors were added, and the DNA library was constructed following the PacBio manufacturing protocols. Next, the library was sequenced using the PacBio Sequel II platform in circular consensus sequencing (CCS) mode following the manufacturer’s instructions. As a result, we obtained a total of 49.17 Gb (~ 83X) PacBio HiFi reads. The average and N50 lengths of the HiFi reads were 14.00 Kb and 14.23 Kb, respectively.

### Genome assembly and annotation

**Fig. 1:**
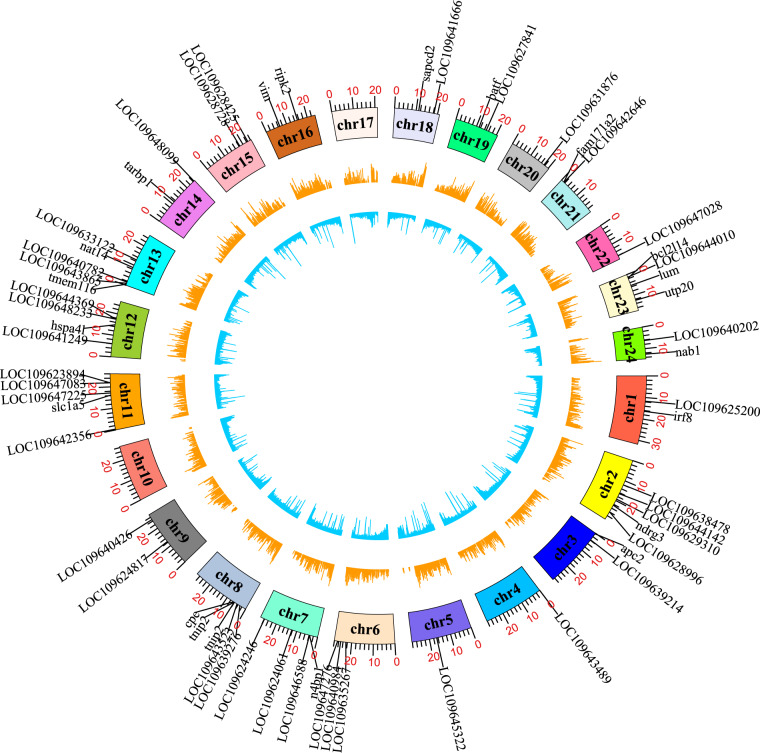
A circos plot of 24 chromosomes of *P. olivaceus* genome. The tracks from inside to outside are: the distributions of transposable element, bar plot for gene density profile, 24 chromosomes, tag labels for candidate key hub genes related to various stresses.

To obtain high-quality Japanese flounder genome sequences, 49.17 Gb PacBio Hifi reads were generated and assembled by Hifiasm (v0.15.1)^24^ with default parameters. After removing redundant sequences using purge_dups (v1.2.5)^25^, a total of 588.22 Mb genome sequences were obtained, and the length of contig N50 was 24.35 Mb, including 46 contigs. Then, RagTag (v2.1.0)^26^ with default parameters was used to scaffold 46 contigs into 25 scaffolds, and therein, 24 chromosome-length scaffolds (Fig. 1) covered 99.99% of the assembled genome sequences, demonstrating a much higher quality genome assembly compared to other published Japanese flounder genomes^7,8^ (Table 1). For genome annotation, we first used EDTA (v2.00)^27^ (--sensitive 1, --anno 1) to annotate genome transposable elements. Approximately 105.88 Mb of repetitive sequences were identified, as shown in Fig. 1, which is much higher than that in previously reported Japanese flounder genome (Table 2). Then, we used Liftoff (v1.6.1)^28^ to map the gene structure annotation of *P. olivaceus* from Refseq (GCF_001970005.1) onto our newly assembled genome. Liftoff used minimap2 to align the gene sequences from Refseq annotation of *P. olivaceus* to the newly assembled genome. On the premise of ensuring transcript and gene structure, Liftoff found the optimal alignment of exons. Then Liftoff integrated information to map existing annotations to the newly assembled genome and generated annotation file. Finally, a total of 22,565 protein-coding genes were identified. The distributions of these genes on each chromosome are shown in Fig. 1.

**Table 1:** Comparative statistic of the *P. olivaceus* genome assembly with old ones.

Genome assembly	This study	Shao *et al*.^8^	Wei *et al*.^7^
Scaffold N50 (Mb)	26.05	23.21	10.55
Contig N50 (Mb)	24.35	0.031	0.036
Total scaffold number	25	6,969	9,525
Total contig number	46	38,614	32,584
Total length (Mb)	588.22	545.78	643.91
GC Content (%)	41.54	41.44	41.64

**Table 2:** Comparative statistic of the *P. olivaceus* repeat sequences with old ones.

Class		This study		Shao *et al*.^8^		Wei *et al*.^7^	
		Length (bp)	% in Genome	Length (bp)	% in Genome	Length (bp)	% in Genome
LTR	Copia	112,330	0.02%	—	—	—	—
	Gypsy	3,786,094	0.64%	975,109	0.18%	237,541	0.05%
	unknown	17,000,615	2.89%	4,362,453	0.82%	3,202,793	0.61%
TIR	CACTA	23,074,608	3.92%	21,956,463	4.11%	22,514,061	4.32%
	Mutator	10,131,242	1.72%	6,863,910	1.28%	5,790,854	1.11%
	PIF_Harbinger	2,410,258	0.41%	1,888,730	0.35%	1,688,125	0.32%
	Tc1_Mariner	845,440	0.14%	930,093	0.17%	427,982	0.08%
	hAT	4,617,190	0.78%	2,578,715	0.48%	2,368,980	0.45%
	polinton	74,664	0.01%	—	—	14,991	0.00%
nonLTR	DIRS_YR	12,352	0.00%	—	—	—	—
	LINE_element	2,852,254	0.48%	787,838	0.15%	718,077	0.14%
nonTIR	helitron	7,640,001	1.30%	4,848,794	0.91%	4,868,953	0.93%
	repeat_region	33,330,298	5.67%	8,190,805	1.53%	8,657,096	1.66%
Total		105,887,346	18.00%	53,382,910	9.99%	50,489,453	9.68%

### Gene coexpression network construction and module-trait association analysis

To identify key genes and modules in response to various stresses by GCN analysis in Japanese flounder, we collected all of the published stress-related RNA-seq datasets, including 16 independent experiments from 3 distinct stresses (i.e., chemical, heat and pathogen stress) encompassing a total of 198 samples (Table 3), until March 31, 2022 from the NCBI SRA database. RNA-seq datasets downloaded in SRA format were first converted into FASTQ format utilizing the fastq-dump tool in SRAtoolkit (v3.0.0)^29^. Then, all sequenced reads were mapped to the high-quality Japanese flounder genome using STAR (v2.7.10a_alpha_220314)^30^ software with default parameters. Next, gene expression data for all genes normalized in transcripts per million (TPM) were calculated by TPMCalculator (v0.0.2) (-q 1)^31^ with sorted bam files obtained from read alignment. Next, the gene expression data were preprocessed using BioNERO (v1.4.2)^32^ based on the following steps: I) displacing missing values (NAs) with 0 utilizing the replace_na function; II) eliminating the genes with average gene expression less than 1 using the remove_nonexp function; III) removing outlying samples using the ZKfiltering function; and IV) adjusting for confounding artifacts using the PC_correction function to make every gene follow an approximate normal distribution. After step-by-step data preprocessing, we obtained a normalized gene expression matrix comprising 13,138 genes whose medial expression values were ≥ 1 from 184 RNA sequencing samples.

**Table 3:** Overview of the RNA-seq datasets used in this study.

Stress		SRA Study	Number of Experiments	Number of Individuals	Platform (Illumina)	Size (GB)	References
Chemicals	bisphenol S- and benzo[a]pyrene	SRP126397	8	22	HiSeq 3000	55.69	^3^
	nickel and cobalt	SRP238940	6	180	HiSeq 2000	48.42	^5^
	contaminated sediment	SRP313148	4	200	HiSeq 2500	91.86	^9^
	copper yrithione and zinc pyrithione	SRP342241	5	650	Hiseq 3000	29.22	^10^
Heat	—	SRP266623	12	36	HiSeq X Ten	249.20	^11^
		SRP330366	12	30	NovaSeq 6000	94.97	^12^
		SRP317999					
		SRP330365					
		SRP324580					
Pathogens	*Edwardsiella tarda*	SRP109689	10	130	HiSeq 4000	143.49	^15^
		PRJNA796380 Un-published	6	—	NovaSeq 6000	—	^43^
		SRR8293419	6	18	Hiseq 4000	125.8	^18^
		SRR8290049					
		SRR8284024					
		SRR8271825					
		SRR8270369					
		SRR8269382					
	*Hirame novirhabdovirus*	SRP162413	2	—	HiSeq X Ten	14.58	—
		SRP162514	4	320	HiSeq X Ten	27.96	^16^
		SRP299209	18	18	HiSeq 2500	117.18	^44^
	*Vibrio anguillarum*	SRP214405	18	18	HiSeq 4000	202.72	^6^
	*Megalocytivirus*	SRP297629	18	72	HiSeq 4000	264.17	^17^
	*Viral hemorrhagic septicemia virus*	SRP102673	36	36	HiSeq 2500	202.76	^45^
		SRP105302	36	40	HiSeq 2500	202.58	^14^
Total	—	—	168		—	1870.6	

**Fig. 2:**
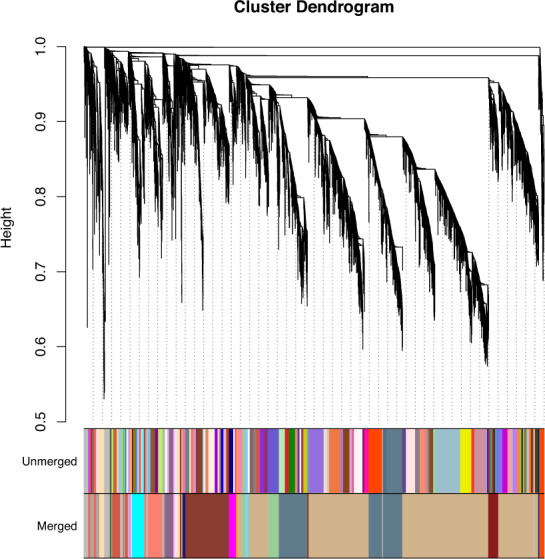
Cluster dendrogram of genes and modules. The branches and color bands represent the assigned module. The tips of the branches represent genes.

Next, the gene coexpression network (GCN) shown in Fig. 2 was constructed using BioNERO with normalized gene expression matrix as input. To make the GCN satisfy the scale-free topology, of which the scale-free topology fit index (R^2^) reaches 0.8 and the mean connectivity tends to 0, we identified the best β power of 9 with the function of SFT_fit. Then, the GCN was inferred using the exp2gcn function with power 9. Eventually, as shown in Fig. 2, a total of 25 coexpression modules were identified, of which the number of genes per module varied from 31 (pink) to 5,947 (tan).

**Fig. 3:**
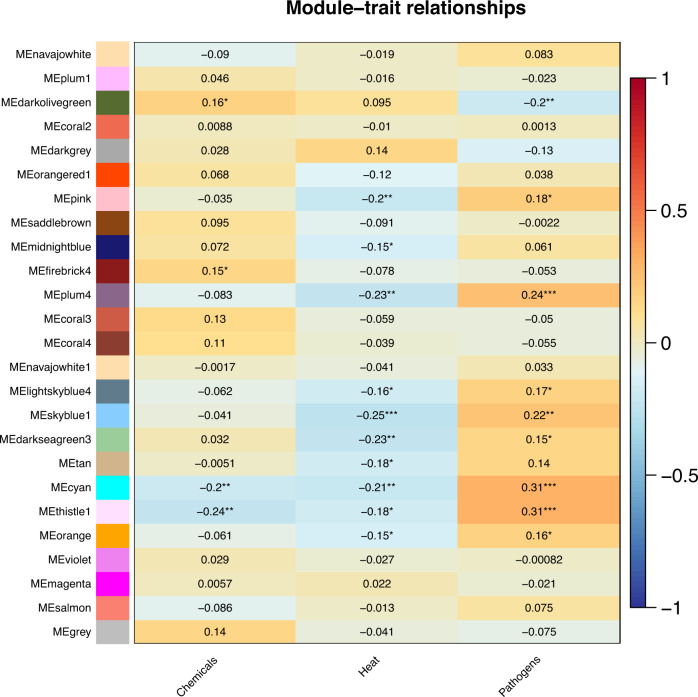
Correlation between modules and stresses. The value in the box is the correlation coefficients. Correlation coefficients with ** or *** represent extremely significant correlation and significant correlation with *.

To identify modules in response to various stresses, the module-trait Spearman correlation coefficients were calculated with the function module_trait_cor in the BioNERO software package. Modules extremely significantly (*p value* < 0.01) positively or negatively correlated with particular traits (stresses) were found, among which 2, 5 and 5 modules were extremely significantly related to chemical, heat, and pathogen stress, respectively. The results are shown in Fig. 3. Interestingly, among these extremely significant modules, one module, the cyan module, was extremely significantly associated with all three distinct stresses. All of these results provide abundant resources for further study of stress-responsive molecular mechanisms in Japanese flounder.

### Module function enrichment analysis

To detect biological processes or pathways to which the genes in the modules that were extremely significantly associated with each stress were related, GO and KEGG function enrichment analyses were conducted using TBtools (v1.09852)^33^. Significantly enriched GO terms and KEGG pathways (corrected *p value* (BH method) <0.5) were identified based on a hypergeometric cumulative distribution function test.

For chemical stress, GO function enrichment results demonstrated that cellular response to chemical stress, cellular response to chemical stimulus, and response to toxic substance were the significantly enriched terms in modules that were extremely significantly related to chemical stress. In addition, KEGG function enrichment analyses showed that chemical carcinogenesis-receptor activation, various signaling pathways related to environmental information processing (such as JAK-STAT, MAPK, and NF-kappa), and signal transduction were significantly enriched pathways in the same modules.

For heat stress, GO function enrichment results illustrated that cellular response to heat, heat shock protein binding, response to heat, lipid catabolic process and catalytic activity were the significantly enriched terms in modules that were extremely significantly correlated with heat stress. Furthermore, KEGG enrichment analyses were conducted on the same modules, and the results showed that cortisol synthesis and secretion and multiple signaling pathways related to environmental information processing (such as NOD-like receptor, JAK-STAT, NF-kappa B, and MAPK) were significantly enriched pathways.

For pathogen stress, GO function enrichment results illustrated that regulation of response to biotic stimulus, immune response, defense response, regulation of immune system process, T-cell activation and alpha-beta T-cell differentiation were the significantly enriched terms in modules that were extremely significantly associated with pathogen stress. Furthermore, KEGG functional enrichment analyses indicated that the immune system, antigen processing and presentation, a variety of signaling pathways (TNF, Toll-like receptor, NOD-like receptor, NF-kappa B, B-cell receptor, T-cell receptor, chemokine, MAPK, etc.), CD molecules and phagosomes were significantly enriched pathways.

It is worth emphasizing that the cyan module, which was extremely significantly associated with all three kinds of stresses, was also significantly enriched in stress-related GO terms, such as stress-activated MAPK cascade, regulation of stress-activated MAPK cascade, regulation of response to stress, p38 MAPK cascade, response to external stimulus, ERK1/2 cascade, and regulation of JNK cascade. Meanwhile, KEGG enrichment results indicated that the cyan module was mainly enriched in environmental information processing-related pathways, such as the MAPK/AMPK/p53 signaling pathway and signal transduction. These results suggested that the cyan module was an important module responsive to different stresses and is worth further study.

### Identification of key hub genes

In this study, candidate key hub genes were defined as the intersections of the hub gene set and differentially expressed gene (DEG) set related to the same stress. First, we established the hub gene set. According to BioNERO’s algorithm, hub genes were selected as the top 10% of genes with the highest kWithin (i.e., the degree of connectivity of the edge of one gene to all other genes under the same module) whose module membership (MM) (i.e., correlation of a gene to its module eigengene) >0.8. The function get_hubs_gcn was used to identify hub genes in each module. Next, hub genes contained in modules extremely significantly associated with the same stress were selected as hub gene set for this stress. Second, the DEG set was established. The featureCounts (-p)^34^ software program in the Subread (v2.0.3)^35^ package was first used to build read count matrixes. Next, edgeR (v3.14)^36^ software was used to identify DEGs with the criteria of false discovery rate (FDR) <0.05 and |log_2_ (fold change) (FC)| > 2. DEGs associated with the same stress were merged into the DEG set for this stress. Finally, 5, 4 and 58 candidate key hub genes associated with chemical, heat, and pathogen stress were identified, respectively. The positions of these key hub genes on chromosomes are shown in Fig. 1.

## Data Records

The raw PacBio sequencing data of the Japanese flounder genome was deposited at the NCBI SRA database under SRA study accession number SRP378268^37^. The final assembled Japanese flounder genome has been submitted to NCBI under accession number GCA_024713975.1^38^. The final genome was also submitted to the National Genomics Data Center with accession number GWHBJWM00000000^39^. In addition, the annotation file of the *P. olivaceous* genome assembly, the gene expression matrix used for GCN construction, and results from GCN analysis including genes per module, DEGs set, hub genes set, GO and KEGG function enrichment, and candidate key hub genes, have been deposited in Figshare^40^.

## Technical Validation

### Completeness and quality assessment of the Japanese flounder genome

Two independent methods were used to assess genome assembly completeness and quality. We first employed Benchmarking Universal Single-Copy Orthologs (BUSCO v5.3.2)^41^ with the actinopterygii_odb10 database, including 3,640 BUSCOs, to evaluate the completeness of the final assembled genome. BUSCO analysis indicated that 98.5% complete BUSCOs were captured, including 97.6% single-copy and 0.9% duplicated BUSCOs. Then, we used Inspector (v1.0.1)^42^ to estimate the assembly quality by mapping PacBio HiFi reads to the assembled contigs to generate read-to-contig alignment and conduct downstream assembly evaluation. The results revealed that the quality value (QV) and read-to-contig mapping rate were 44.31 and 99.91%, respectively. The above evaluation results demonstrated the high quality and completeness of the reported genome assembly, which will contribute to further research on the genomics and genetics of Japanese flounder.

## Data Availability

The data analysis methods, software and associated parameters used in present study are described in the section of Methods. If no detail parameters were described for software used in this study, default parameters were employed. No custom scripts were generated in this work.
